# A mix of S and ΔS variants of STAT3 enable survival of activated B-cell-like diffuse large B-cell lymphoma cells in culture

**DOI:** 10.1038/oncsis.2015.44

**Published:** 2016-01-04

**Authors:** M Zheng, K B Turton, F Zhu, Y Li, K M Grindle, D S Annis, L Lu, A C Drennan, D J Tweardy, U Bharadwaj, D F Mosher, L Rui

**Affiliations:** 1 Division of Hematology-Oncology, Department of Medicine, Carbone Cancer Center, School of Medicine and Public Health, University of Wisconsin-Madison, Madison, WI, USA; 2 Department of Hematology, Tongji Hospital, Tongji Medical College, Huazhong University of Science and Technology, Wuhan, China; 3 Department of Biomolecular Chemistry, School of Medicine and Public Health, University of Wisconsin-Madison, Madison, WI, USA; 4 Department of Internal Medicine, MD Anderson Cancer Center, Houston, TX, USA

## Abstract

Activated B-cell-like diffuse large B-cell lymphoma (ABC DLBCL) is characterized by increased expression and activator of signal transducer and activator of transcription 3 (STAT3). ABC DLBCL cells require STAT3 for growth in culture. In ABC DLBCL cells, eosinophils and perhaps all cells, four variant STAT3 mRNAs (Sα, ΔSα, Sβ and ΔSβ) are present as a result of two alternative splicing events, one that results in the inclusion of a 55-residue C-terminal transactivation domain (α) or a truncated C-terminal domain with 7 unique residues (β) and a second that includes (S) or excludes (ΔS) the codon for Ser-701 in the linker between the SH2 and C-terminal domains. A substantial literature indicates that both α and β variants are required for optimal STAT3 function, but nothing is known about functions of ΔS variants. We used a knockdown/re-expression strategy to explore whether survival of ABC DLBCL cells requires that the four variants be in an appropriate ratio. No single variant rescued survival as well as STAT3Sα-C, Sα with activating mutations (A661C and N663C) in the SH2 domain. Better rescue was achieved when all four variants were re-expressed or Sα and ΔSα or Sβ and ΔSβ were re-expressed in pairs. Rescue correlated with expression of STAT3-sensitive genes NFKBIA and NFKBIZ. We consider a variety of explanations why a mix of S and ΔS variants of STAT3 should enable survival of ABC DLBCL cells.

## Introduction

Signal transducer and activator of transcription 3 (STAT3), a transcription factor in the Janus kinase (JAK)/STAT signaling pathway, is positioned at the crossroads between immunity and malignancy.^1, 2^ Activity of STAT3 is tightly regulated with a transient activation during the normal immune response, whereas it maintains a constitutively activated status in many solid and hematological cancers.^3, 4, 5^ In diffuse large B-cell lymphoma (DLBCL), STAT3 is overexpressed and persistently activated in the activated B-cell-like (ABC) subtype but not in the germinal center B-cell-like (GCB) subtype.^6, 7, 8^ Constitutive activation of STAT3 results from autocrine production of the cytokines IL-6 or IL-10, which is caused by MYD88 mutations and NF-κB activation.^9, 10^ Autocrine activation of STAT3 is required for tumor growth of ABC DLBCL,^11^ presumably by increasing transcription of disease-specific genes that promote cell proliferation and survival, such as NFKBIZ.^12, 13^

STAT3 is activated by phosphorylation of Tyr-705, which can be catalyzed by JAKs working downstream of cytokine or growth factor receptors and by several non-receptor tyrosine kinases.^1, 14^ Phosphorylated STAT3 homodimerizes through reciprocal phospho-tyrosine–SH2 domain interactions, then translocates to the nucleus and binds to cognate elements on the promoters of responsive genes. Phosphorylation of Thr-714 and Ser-727 is also required for optimal transcriptional activity.^15, 16^ STAT3 has two well-characterized splice variants, STAT3α and β, because of alternative splicing that results in a 55-residue transactivation domain (α) or truncation of the domain with 7 unique C-terminal residues (β).^17, 18, 19^ Consistent with the absence from STAT3β of most of the C-terminal transactivation domain and Ser-727, initial biochemical analyses suggested that STAT3β blocks the transcriptional function of the STAT3α protein in a dominant-negative manner.^18^ A gene-targeting mouse study, however, did not support this conclusion, demonstrating that STAT3β expression can rescue the embryonic lethality of a complete STAT3 deletion and activate specific STAT3 target genes.^20^ Despite functional overlap between the two variants, STAT3α also was shown to have non-redundant roles in modulation of cellular responses to IL-6 or IL-10.^20^

The existence of the α and β splice variants may not totally account for functional heterogeneity of STAT3. There are two other splice variants, STAT3ΔSα and ΔSβ, which are a result of a second splicing event that includes (S) or excludes (ΔS) the codon for Ser-701 in the linker between the SH2 and C-terminal domains.^21^ We detected mRNAs of the ΔS variants in both eosinophils and ABC DLBCL cells and found comparable splice variant ratios (Sα ~75%, Sβ ~12%, ΔSα ~10% and ΔSβ ~3%) despite differences in total levels of STAT3 transcripts in the two types of cells.^21^ There was a tendency for the β splicing event to be paired with the ΔS splicing event, indicating that the two events are not completely independent. Analysis of publicly-available RNA-Seq data of 16 human tissues (GEO accession GSE30611) revealed that the ΔS variants account for 10–26% of the total,^21^ in accord with a prior investigation of tandem alternate donor splicing in which STAT3's ΔS proportion was consistently around 17% of total in human and mice leukocytes.^22^ Thus, although ΔS STAT3 variants are less abundant than S, the ΔS/S ratio remains relatively constant in tissues.^21^ Further, the ratio is conserved among species.^22^ These findings suggest that ΔS/S splicing is functionally indispensible, that is, function of STAT3 depends on having a mix of proteins that have or lack Ser-701.

Here, we performed functional analysis of the four STAT3 variants in STAT3-dependent ABC DLBCL cells. We used a knockdown/re-expression strategy to examine whether individual variants or their different combinations reversed the toxicity of shRNAs that selectively target endogenous STAT3. The results showed that no single variant rescued survival as well as STAT3Sα-C, an oncogenic Sα variant with activating mutations in the SH2 domain.^23^ Better rescue was achieved when all four variants were re-expressed or Sα and ΔSα or Sβ and ΔSβ were re-expressed in pairs. Rescue correlated with expression of STAT3-sensitive genes NFKBIA and NFKBIZ. These results indicate that the mix of S/ΔS splicing is indeed required for optimal STAT3 function.

## Results

### Use of a knockdown and re-expression strategy for functional analysis of STAT3 splice variants

**Figure 1:**
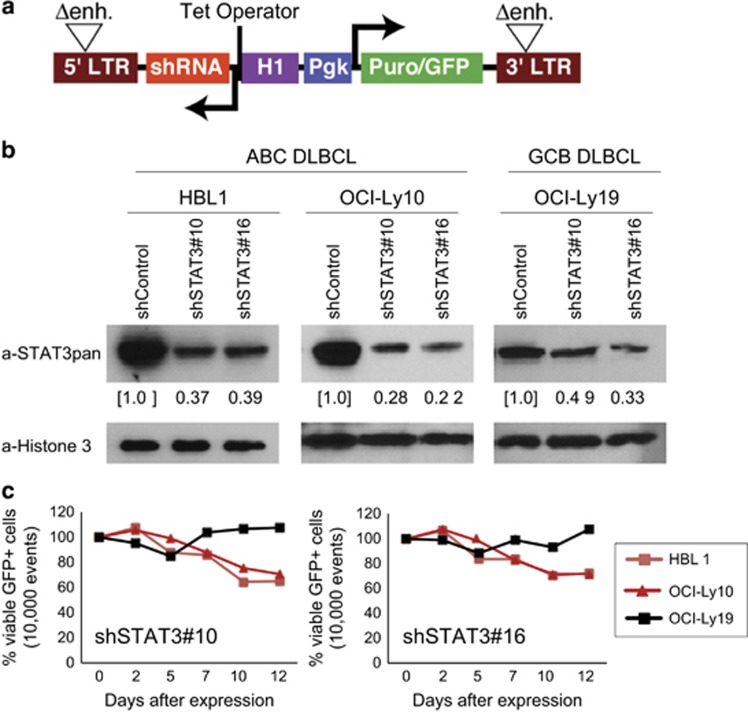
Knockdown of total endogenous STAT3 with shRNA#10 and shRNA#16. (**a**) The shRNA retroviral vector constructs. Two tetracycline repressor binding sites (Tet operators) were inserted into the histone H1 promoter, which drives shRNA expression. LTR, long terminal repeat; Pgk, phosphoglycerate kinase promoter; puro^r^/GFP, fusion of puromycin resistance and green fluorescent protein gene; Δenh., deletion of LTR promoter sequences. (**b**) Characterization of cells expressing shSTAT3#10, shSTAT#16 or shControl. Three days after shRNA induction, STAT3 knockdown efficiency was analyzed by immunoblotting in ABC DLBCL (HBL1 and OCI-Ly10) and GCB DLBCL (OCI-Ly19) cell lines. Reduced expression of STAT3 by shRNAs relative to the control shRNA as quantified by densitometry. A level of expression was first normalized to that of the individual histone H3 loading control and then to that in the control shRNA. (**c**) Selective toxicity of STAT3 knockdown by shRNAs for ABC DLBCL cells. The percentage of viable GFP+ shSTAT3 expressing cells was normalized to that of the control shRNA for each time point.

To develop a system to test functionality of the four STAT3 variants, we used two representative ABC DLBCL cell lines, OCI-Ly10 and HBL1, in which high activity of STAT3 is driven by the autocrine cytokines IL-6 or IL-10 and promotes cell survival and proliferation.^6, 11, 24^ The GCB cell line OCI-Ly19 with no constitutive STAT3 activation served as a control. To re-evaluate a survival role for STAT3, we generated two small hairpin (sh) RNAs against the common 3'UTR for all four splice variants and retrovirally transduced the shRNAs into these lymphoma cells along with the marker GFP; the percentage of GFP+ cells was calculated by flow-cytometric analysis during 12 days of observation (Figure 1a). The results demonstrated that either shRNA knocked down endogenous STAT3 by >60% (Figure 1b) and selectively reduced cell viability of cultured ABC DLBCL as compared with GCB DLBCL cells (Figure 1c). To build upon this finding, we engineered the tetracycline-inducible system into the three cell lines in which shRNA expression is controlled by the Tet repressor, allowing growth and selection of cells for exogenous expression of STAT3 variants before shRNA-mediated suppression of endogenous STAT3 expression.

**Figure 2:**
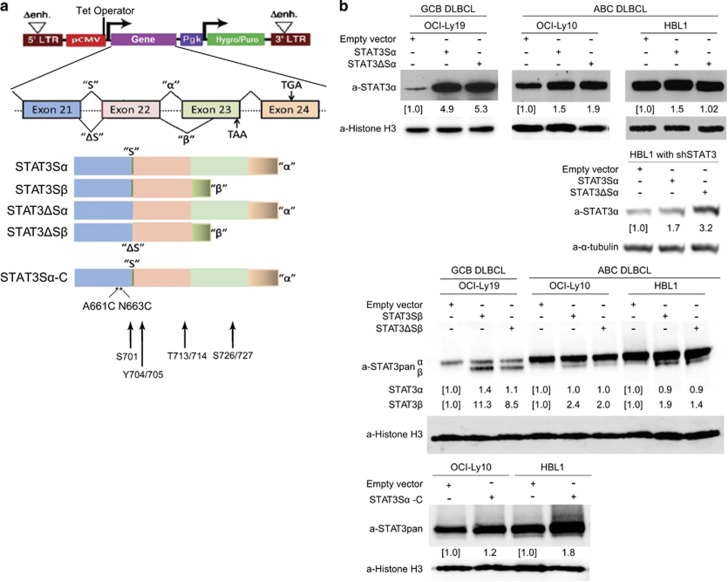
Expression of single STAT3 splice variants. (**a**) Schematics of the retroviral vector constructs of single STAT3 variants (hygromycin resistance) and constitutively active STAT3Sα-C (puromycin resistance). Two tetracycline repressor binding sites (Tet operators) were inserted into CMV promoter, which drives STAT3 variant expression. Hygro/puro, hygromycin or puromycin resistance gene; LTR, long terminal repeat; Pgk, phosphoglycerate kinase promoter; Δenh., deletion of LTR promoter sequences. Arrows indicate STAT3 phosphorylation sites, S701, Y704/705, T713/714 and S726/727. (**b**) OCI-Ly19, OCI-Ly10 or HBL1 cells were transduced with Sα, ΔSα, Sβ or ΔSβ constructs, whereas STAT3Sα-C was expressed in OCI-Ly10 and HBL1 cells. Three days after induction, expression levels of these constructs were analyzed by immunoblotting with STAT3 antibodies that recognize only Sα and ΔSα (top and bottom panels) or all four variants (middle panel). Note that protein levels include expression of endogenous STAT3 except for one experiment in which endogenous STAT3 was knocked down with 3 days of expression of shSTAT3#10 in HBL1 cells. Numbers indicate expression of STAT3 variants relative to the EV control as quantified by densitometry.

Our recent study revealed that all four variant STAT3 mRNAs (Sα, ΔSα, Sβ and ΔSβ) are present in OCI-Ly10 and HBL1 cells, with a similar expression pattern to eosinophils.^21^ To generate viable stably expressing cell lines, we cloned individual variants into a selectable retroviral vector (Figure 2a). The vector lacks the 3′UTR of STAT3, so exogenous expression of these variants would not be affected by the STAT3 shRNAs. In parallel, as a possible positive control, we used the constitutively activated form of STAT3 (STAT3Sα-C), the α variant with activating mutations (A661C and N663C) in the SH2 domain.^23^ STAT3Sα-C can form a dimer driven by oxidation of cysteines rather than by phosphorylation of Tyr-705,^23^ and has been shown to cause transformation of fibroblasts and protect fibroblasts against apoptosis.^23, 25^ After antibiotic selection, expression of STAT3 in the retroviral transduced stable cell lines was verified by immunoblotting analysis with antibodies that recognize either α and ΔSα or all four variants. After 3 days of induction, all the individual variants were expressed but at various levels when compared with endogenous levels as shown in the empty vector (EV) control (Figure 2b). In HBL1 cells, an increase in ΔSα expression that was retrovirus mediated was limited. However, when the high level was eliminated by knockdown of endogenous STAT3, a more than threefold increase in ΔSα expression was achieved (Figure 2b).

### Insufficient functional rescue by the single STAT3 variants

**Figure 3:**
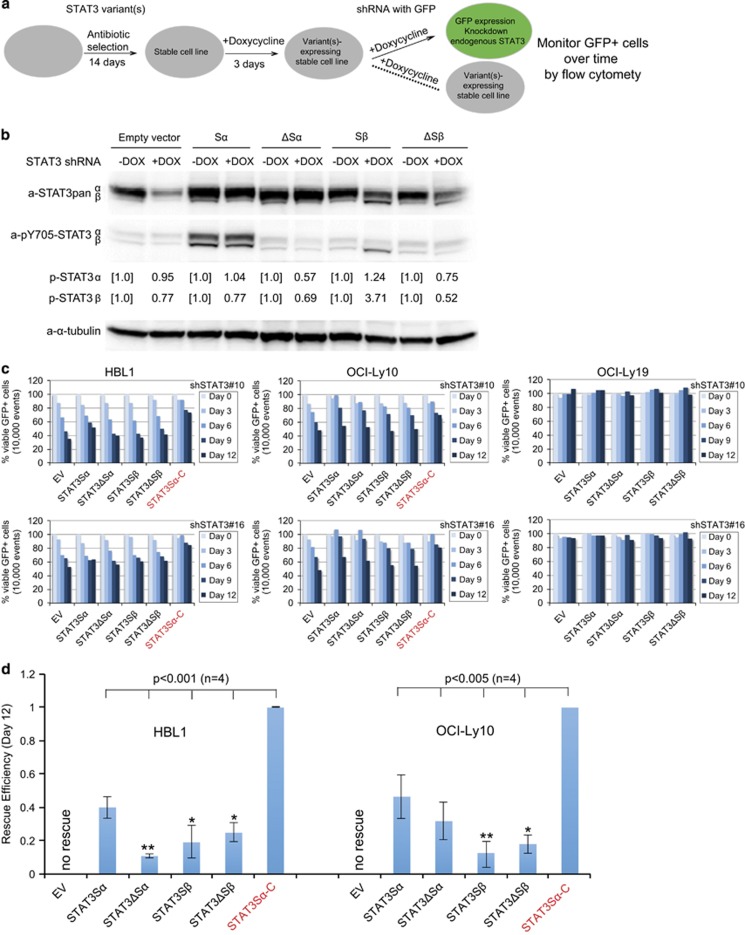
Rescue experiments using single STAT3 variants. (**a**) Workflow of knockdown/re-expression approach. Different stable cell lines were induced for expression of their individual STAT3 variants or EV control for 3 days before transduction with STAT3 shRNAs with the marker GFP. The shRNA-expressing GFP+ cells were monitored by flow cytometry over 12 days of observation. (**b**) Expression and activation of the single STAT3 variants in ABC DLBCL cells after knockdown of endogenous STAT3 by shRNA#16. OCI-Ly10 cells were manipulated as described in **a**, and shSTAT3 cells were selected with puromycin. After 5 days of selection, shSTAT3 was induced for expression for 4 days before immunoblotting with STAT3 or phospho-tyrosine 705 (pY705) antibody. Note that this monoclonal antibody did not recognize phospho-tyrosine 704 of STAT3ΔSα and ΔSβ. Expression of pY705 STAT3α or β relative to the uninduced control was quantified by densitometry. (**c**) Insufficient rescue by the individual variants. ABC DLBCL cell lines HBL1 and OCI-Ly10 and control GCB cell line OCI-Ly19 were used following the experimental procedure as described in **a**. The percentage of GFP+ cells expressing STAT3 shRNAs was normalized to that of the control shRNA at each time point. STAT3Sα-C expressing cells served as positive controls. The experiments were repeated twice with two different STAT3 shRNAs. (**d**) The rescue efficiencies of different STAT3 variant stable cell lines on day 12 were calculated by a formula: (% variant−% EV)/(% STAT3Sα-C−% EV). Error bars represent mean±s.d. (*n*=4, *P<0.05, **P<0.01, when compared with STAT3Sα).

We then knocked down endogenous STAT3 by shRNA in these cell lines to see which variant can reverse the shRNA-mediated toxicity. As illustrated in Figure 3a, the stable cell lines were induced for expression of the variants for 3 days before transduction of STAT3 shRNAs, and cultures were monitored beginning 3 days after that for percentage of cells positive for GFP as a marker of shRNA expression. Dual expression of STAT3 variants and shRNAs was forced over 12 days with periodic flow-cytometric analysis (Figure 3a). Immunoblotting analysis in which STAT3Sα and STAT3Sβ were distinguished by mobility demonstrated that expected levels of retrovirus-mediated expression of each variant were maintained after knockdown of endogenous STAT3 by shRNA when compared with that in the EV control cells (Figure 3b). Because of auto-secretion of cytokines by the lymphoma cells,^6, 9^ it should be possible to activate expressed variant STAT3 and thus replace activation of endogenous STAT3s. Both Sα and Sβ variants reacted with the D3A7 monoclonal anti-phospho-Tyr-705 antibody, with reactivity with Sβ being greater than with Sα (Figure 3b). ΔSα and ΔSβ variants were not recognized by the phosphosite-specific antibody. Two possibilities are that the ΔS variants are not phosphorylated or that Ser-701 is a required part of the epitope for the phosphosite-specific antibody. Because a peptide has been detected in a global analysis of the liver phosphoproteome in which Tyr-704 is phosphorylated in the absence of Ser-701,^26^ we favor the second possibility.

Oncogenic STAT3Sα-C rescued the toxicity of two independent STAT3 shRNAs in two ABC DLBCL cell lines HBL1 and OCI-Ly10, as evidenced by over 80% shRNA-expressing cells remaining in culture after 12 days of shRNA induction (Figure 3c), and thus was considered as a positive control with which to measure rescue by splice variants lacking the oncogenic mutation according to the formula: (% variant−% EV)/(% STAT3Sα-C−% EV). No single variant was sufficient to prevent STAT3 shRNA-mediated cell loss from the culture (Figures 3c and d). STAT3Sα had the best rescue efficiency of the four variants, but still less than half that of STAT3Sα-C (Figure 3d). These results suggest that STAT3Sα, despite being the most abundant variant, has a limited role on its own in promoting survival of ABC DLBCL cells, and STAT3 requires more than one variant for optimal function.

### A mix of S and ΔS variants of STAT3 rescue STAT3 shRNA-mediated cell death

**Figure 4:**
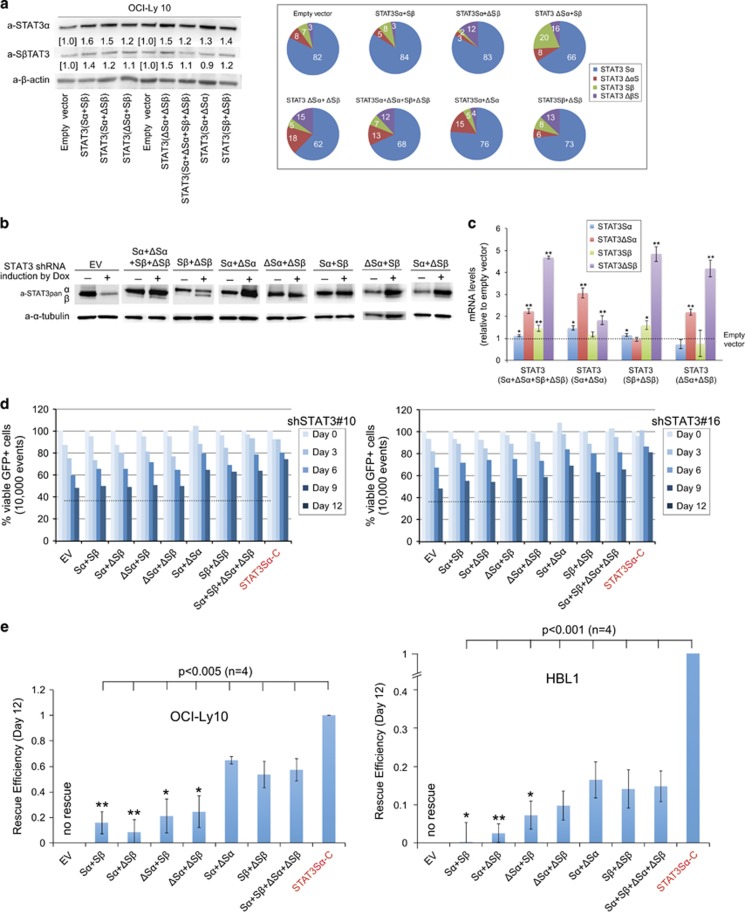
Rescue experiments using combinations of STAT3 variants. (**a**) Verification of expression of combinatorial STAT3 variants in OCI-Ly10 cells after 3 days of induction by immunoblotting with specific antibodies to STAT3α and β (left panel) or qPCR (right panel) (see Materials and Methods for details). It should be stressed that neither method distinguishes between exogenously and endogenously expressed STAT3 variants. (**b**, **c**) Verification of expression of combinatorial STAT3 variants in OCI-Ly10 cells after 3 days of induction by immunoblotting (**b**) or qPCR (**c**) when endogenous STAT3 variants were knocked down by STAT3 shRNA. (**d**) Partial rescue by re-expression of Sα and ΔSα or Sβ and ΔSβ or together all four variants. ABC DLBCL cell lines HBL1 and OCI-Ly10 were used following the experimental procedure as described in **a**. The percentage of GFP+ cells expressing STAT3 shRNAs was normalized to that of the control shRNA at each time point. STAT3Sα-C expressing cells served as positive controls. The experiments were repeated twice with two different STAT3 shRNAs. (**e**) The rescue efficiencies of different combinatorial STAT3 variant stable cell lines on day 12 were calculated by a formula: (% variant−% EV)/(% STAT3Sα-C−% EV). Error bars represent mean±s.d. (*n*=4, *P<0.05, **P<0.01, when compared with a pair of Sα and ΔSα).

Given the insufficient rescue of STAT3 shRNA by any individual variant, we asked whether a combination of variants could increase rescue efficiency. We established stable cell lines in which variants were co-expressed in pairs or all variants were expressed. Protein and mRNA expression was estimated by immunoblotting and quantitative PCR after induction with doxycycline (Figure 4a). In addition to the antibody specific for STAT3α, we used the monoclonal antibody recently developed to detect STAT3β.^27^ The result demonstrated that for the most part the expected protein levels were elevated after co-expression of these variants when compared with the EV (Figure 4a, left panel). However, expression of only the STAT3β variants was associated with a concomitant increase in STAT3Sα. Consistent with our previous quantitative RT–PCR (qPCR) analyses,^21^ a bias in endogenous STAT3 splice variant ratios in the EV control was observed; that is, α was more abundant than β, and S was more common than ΔS (Figure 4a, right panel). Again, the ratios for the most part changed in the directions predicted except that the proportion of STAT3Sα was maintained when only STAT3Sβ and STAT3ΔSβ were exogenously expressed.

We next measured levels of protein and mRNA when combined variants were expressed as endogenous STAT3 was being knocked down by shRNA. Immunoblotting analysis confirmed expected exogenous expression of these variants (Figure 4b). Consistently, quantitative PCR data showed that levels of expression were significantly higher than that of the EV control (Figure 4c). Similar to the results in Figure 4a, exogenous expression of a pair of Sβ+ΔSβ sustained expression of endogenous Sα. This is likely through a STAT3 self-regulation mechanism, given that Sβ+ΔSβ are a functional combination based on the following rescue assay.

As was done with the attempted rescue with individual variants, the combined variants were induced for expression before knockdown of endogenous STAT3 by shRNA and percent of cells expressing GFP and shRNA were monitored by flow cytometry over 12 days. STAT3Sα-C again served as a positive control, and the EV as a negative control. The results indicated that a combination of Sα and ΔSα, Sβ and ΔSβ, or all four variants rescued STAT3 shRNA-mediated cell death from culture better than the other combinations (Figures 4d and e). Thus, these data indicate functional importance of the ΔS variants, which work together with the S variants to enable survival of ABC DLBCL cells in culture.

### Correlation of rescue efficiency with expression of STAT3 target genes

**Figure 5:**
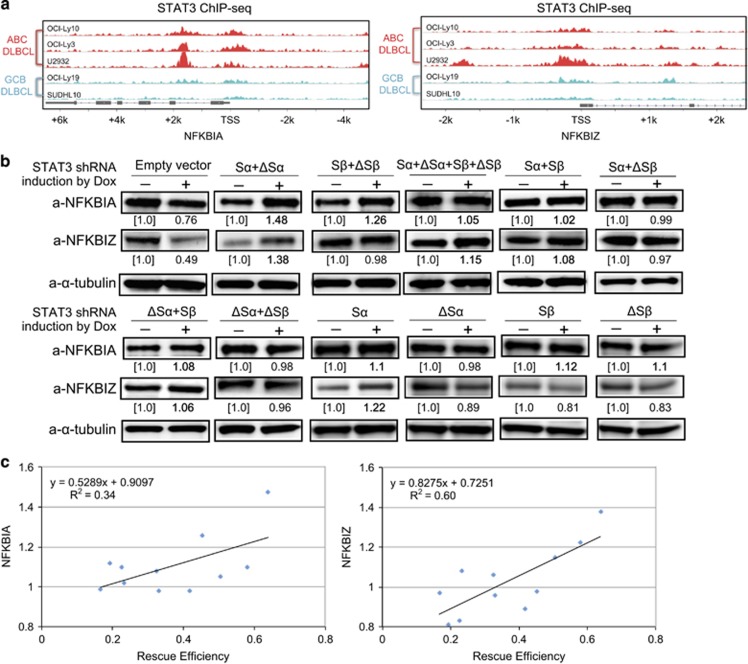
Correlation of rescue efficiency and expression of STAT3 target genes NFKBIA and NFKBIZ. (**a**) Mining of ChIP-seq data of the indicated ABC versus GCB DLBCL cell lines from a recent study^12^ shows that NFKBIA and NFKBIZ are specific STAT3 targets for ABC DLBCL. TSS, transcription start site. (**b**) Immunoblotting analysis of expression of NFKBIA and NFKBIZ in OCI-Ly10 cells after 4 days of induction for expression of the indicated single or combinatorial STAT3 variants. Endogenous STAT3 variants were simultaneously knocked down by shRNA. Numbers represent expression levels relative to the uninduced control as determined by densitometry. (**c**) The correlation analysis shows a positive correlation between rescue efficiency and expression of NFKBIA or NFKBIZ. ‘R' denotes non-parametric Spearman's rank correlation coefficient.

The likely molecular mechanism of rescue is that exogenously expressed STAT3 variants replace transcriptional function of endogenous STAT3s. An important feature of ABC DLBCL is NF-κB activation, which promotes cancer cell proliferation and survival.^28^ Some NF-κB genes in ABC DLBCL are known targets of STAT3, including two important genes, NFKBIA and NFKBIZ.^12, 13^ NFKBIA is a key regulator for both canonical and non-canonical NF-κB activation.^28^ NFKBIZ is an essential gene in ABC DLBCL, as knockdown by its shRNA causes death of cultured cancer cells.^13^ We mined STAT3 ChIP-seq data from a recent study^12^ and found apparent STAT3 occupancy on promoter or body regions of NFKBIA and NFKBIZ in three ABC DLBCL cell lines in comparison with two GCB cell lines (Figure 5a), indicating that NFKBIA and NFKBIZ are specific STAT3 targets in ABC DLBCL.

Therefore, we investigated the expression of NFKBIA and NFKBIZ in the above stable cell lines with or without knockdown of endogenous STAT3. The immunoblotting results demonstrated reduced expression of both NFKBIA and NFKBIZ in EV control cells after STAT3 shRNA induction, which is further evidence that these are STAT3 target genes (Figure 5b). Interestingly, either single variants or a mix of S and ΔS variants were able to sustain expression of NFKBIA and NFKBIZ, but relatively high expression levels were observed in cells that co-expressed S and ΔS variants or a single α variant (Figure 5b).

To examine whether the expression of STAT3 target genes is associated with functionality of the variants, we performed correlation analysis to assess the rescue efficiency and expression levels of NFKBIA and NFKBIZ. The results demonstrated that the rescue efficiency positively correlated with expression of both NFKBIA and NFKBIZ (Figure 5c). Together, these findings indicate that the STAT3 variants depend on their transcriptional activity to enable survival of cultured ABC DLBCL.

## Discussion

STAT3 is best known as forming a homo- or heterodimer for transcriptional activity and functionality.^2, 14^ The Sα and Sβ variants have been well studied, but biology of two ΔS variants (Ser-701 deletion in Sα or Sβ) is unexplored. In this study, we used a knockdown/re-expression strategy to dissect the role of the STAT3 variants in the survival of STAT3-dependent ABC DLBCL cells; that is, whether expression of the individual STAT3 variants or different combinations rescues cell death caused by STAT3 shRNAs that target 3′UTR of endogenous STAT3. We found that any single variant was not sufficient to reverse STAT3 shRNA-mediated cell toxicity, the best being a moderate rescue by Sα, the most abundant variant (~80% in DLBCL). However, expression of a pair of Sα and ΔSα, a pair of Sβ and ΔSβ, or all four variants had a better rescue efficiency. These findings suggest that dimerization with a STAT3ΔS is required for optimal function of STAT3. The rescue mediated by these variants likely depends on their transcriptional activity given a positive correlation between rescue efficiency and expression of STAT3-sensitive genes NFKBIA and NFKBIZ.

The molecular mechanisms underlying functionalities of the variants with Ser-701 deletion are unknown. Recent large-scale proteomics studies may provide insight into the phosphostatus of STAT3 ΔS variants. The stability of phospho-Tyr-704/5 differs in α and β.^4^ Phosphorylation of residue Tyr-705 (Tyr-704 in ΔS variants) accompanies canonical STAT3 activation. Ser-701 can be phosphorylated in the presence or absence of Tyr-705 phosphorylation.^29^ Furthermore, Tyr-704 of STAT3 ΔS splice variants can be phosphorylated,^26^ although as discussed in Results the anti-phospho-Tyr-705 monoclonal antibody we used may have been unable to detect the pTyr-704 epitope (Figure 3). Thus, four different phosphoforms are possible: phosphorylation of Tyr-704 or Tyr-705 alone, of Ser-701 alone, or of Ser-701 and Tyr-705. Although not localized in a STAT3β crystal structure, based on immediately proximal residues Ser-701 is near the dimer interface.^30^ Thus, the four phosphoforms may have different kinetics of dimerization and interaction with DNA. Also, the four phosphoforms may influence two other phosphorylation sites: Ser-727 (present only in STAT3 α variants) and Thr-713/714.^16^ Further investigations will require methods to analyze phosphoform distribution, how the distribution is influenced by different signaling axes and the effects of the phosphoforms on STAT3-modulated transcription.

The rescue efficiency was better upon transducing pairs of splice variants. The best pairs were Sα+Δα and Sβ+Δβ, whereas rescue by Sα+ΔSβ and ΔSα+Sβ was poor (Figure 4). Given the small proportion of ΔS splice variants in the endogenous population, these findings were unexpected. STAT3Sα and Sβ regulate overlapping gene sets, but have certain distinct functions (reviewed in Dewilde *et al.*^31^). STAT3 is able to form α-α, β-β and α-β dimers; and electrophoretic mobility shift assay (EMSA) competition assays suggest that α preferentially dimerizes with β rather than another α, leading to a factor with decreased transcription efficiency.^18^ The significance of STAT3 ΔS splice variants in dimerization has not been investigated. Our data may indicate that in Sα-Sβ combinations, the STAT3 heterodimer level is too high, decreasing transcription efficiency. In a pilot experiment, over-expression of any of the variants with the GFP retroviral vector on top of endogenous proteins was toxic to the lymphoma cells (loss of 30–50% of GFP+ cells from culture), which suggests that maintaining of a natural ratio of STAT3 variants is critical for cell fitness.

The experimental system was limited by inability to control or measure endogenous STAT3 facilely after knockdown. The immunoblotting and qPCR were performed at day 5 post induction, while rescue was assessed at day 12 when STAT3 levels are more likely to be determined by the constructs. Further, we do not know the full panel of genes required for lymphoma cell viability. Expression of any single variant sustained expression of NFKBIA and NFKBIZ after knockdown of endogenous STAT3 (Figure 5b). It is possible that a remaining endogenous variant contributes to transcriptional activity by dimerization with an exogenous variant. We are currently developing an inducible CRISPR/Cas9 system to knockout endogenous STAT3, which perhaps will allow us to explore functionality and transcriptional activity of ΔS variants in a more precise way.

Adding to the complexity, STAT3 can dimerize with other members of the STAT family or even other proteins. STAT3Sβ increases its transcriptional activity by interacting with c-Jun.^17^ STAT3 can dimerize with STAT5a/b^32^ and STAT1.^33^ In ABC DLBCL, the IFN-STAT1 pathway is lethal and thus basal STAT1 activity is low.^34^ Unphosphorylated STAT3 can bind to p65 of NF-κB.^35^ All of these proteins are expressed in ABC DLBCL.^7, 13, 36^ We cannot rule out the possibility of these interactions in DLBCL cells, but dimerization with non-STAT3 proteins seems unlikely to be functionally important, given little or no rescue by any of the single variants of STAT3.

In summary, the data presented here indicate that a pair of S and ΔS variants are required for optimal STAT3 function in ABC DLBCL cells, including maintaining cell survival and activating the target genes NFKBIA and NFKBIZ. This functional analysis offers a starting point for the study of ΔS variants. Further research includes structural assessments of dimerization of the S and ΔS variants and genome-wide analysis of transcriptional activity of the ΔS variants.

## Materials and methods

### Cell lines and culture

Doxycycline-inducible human diffuse large B-cell lymphoma cell lines (HBL1, OCI-Ly10 and OCI-Ly19) that express the bacterial tetracycline repressor were engineered as described previously.^37^ Doxycycline (20 ng/ml) was used for inducing the expression of genes of interest. The cell lines were grown in RPMI-1640 media (Hyclone, Logan, UT, USA) supplemented with 20% FBS (fetal bovine serum, Atlanta Biologicals, Flowery Branch, GA, USA), 100 U/ml penicillin, 100 μg/ml streptomycin (Corning Cellgro, Manassas, VA, USA), 2 mm GlutaGRO (Corning Cellgro), 1 × MEM-NEAA (Quanlity Biological, Inc., Gaithersburg, MD, USA) and 1 mm Sodium Pyruvate Solution (Hyclone). All cultures were routinely tested for mycoplasma contamination. Human embryonic kidney cell line 293 T was cultured in DMEM (Dulbecco's modified Eagle's medium, Hyclone) with 10% FBS. All cell lines were cultured at 37 °C in a 5% CO_2_ atmosphere.

### Plasmid construction, retroviral production and generation of antibiotic-resistant cell lines

STAT3 constructs were amplified from eosinophil RNA by RT–PCR and cloned into pcDNA3.1+ using standard molecular biology techniques. Briefly, eosinophil RNA was isolated, and cDNA was generated using random primers and SuperScript III first-strand synthesis system (Invitrogen Life Technologies, Grand Island, NY, USA).^21^ STAT3 variants, Sα, ΔSα, Sβ and ΔSβ were amplified by PCR and cloned into the *Hin*dIII and *Xho*1 restriction sites of pcDNA3.1+. A silent mutation, encoding for a Spe1 site, at Leu670 was added to aid in downstream sequence manipulation. The complete sequence of inserts was verified before transferring to pRetroCMV/TO vectors, which are inducible CMV/TO retroviral vectors (eGFP negative) with a selection marker hygromycin.^38^ Four full-length STAT3 variants were each subcloned into another inducible CMV/TO retroviral vector (eGFP+) with a selection marker hygromycin.^38^ These retroviral vectors of STAT3 variants were used for subsequent transfection into DLBCL cell lines. The STAT3Sα-C (constitutively active STAT3) plasmid was a gift from the Lou Staudt lab (Bethesda, MD, USA). STAT3 shRNAs were prepared in a doxycycline-inducible retroviral vector with a selection marker puromycin as described previously,^37^ with sequences of shSTAT3 #1 (forward: 5'-GGCAAAGGCTTACTGATAAAC-3', reverse: 5'-GTTTATCAGTAAGCCTTTGCC-3'), shSTAT3 #2 (forward: 5'-GGGCTTACCATTGGGTTTAAA-3', reverse: 5'-TTTAAACCCAATGGTAAGCCC-3'). These STAT3 shRNAs only target total endogenous STAT3 expression, and have no effect on exogenous STAT3.

The ABC DLBCL cell lines HBL1 and OCI-Ly10 and GCB DLBCL cell line OCI-Ly19 were infected by retroviral supernatants. The infected cells were selected with puromycin (1 μg/ml) or hygromycin (200–500 μg/ml) for 2 weeks to generate different stable cell lines or 1 week for selection of shRNA-expressing cells.

### Cell viability assay

Cell viability was measured with an automatic cell counter according to the manufacturer's instructions. Briefly, cells were harvested and suspended, and mixed with equal volume of 0.4% trypan blue. Ten microliters of the cell suspension was loaded onto TC20 system (Bio-Rad, Hercules, CA, USA) counting slides, and the percentage of viable cells was quantified on a TC20 automated cell counter (Bio-Rad).

### Flow cytometry

For analysis of cell survival rate, cells were collected and washed twice with cold phosphate-buffered saline. GFP-positive cells were analyzed with BD AccuriTM C6 (BD Biosciences, San Jose, CA, USA). Data were analyzed by the Flowjo software (BD Biosciences, Ashland, OR, USA).

### Immunoblotting assay

Cells were lysed using MAPK lysis buffer (50 mm HEPES, 4 mm sodium pyrophosphate, 10 mm sodium fluoride, 2 mm orthovanadate, 100 mm NaCl, 10 mm EDTA, pH 7.5) with protease inhibitor cocktail (Sigma, St Louis, MO, USA). Protein concentrations were determined by BCA assay (Thermo Scientific, Grand Island, NY, USA). Proteins were separated by SDS–PAGE and transferred onto nitrocellulose membranes. After blocking with non-fat milk, the membrane was probed with primary antibodies against proteins of interest. The primary antibodies included anti-STAT3pan (124H6, from Cell Signaling Technology, Danvers, MA, USA), anti-STAT3α (9D8, ab119352, from Abcam, Cambridge, MA, USA), anti-STAT3β,^27^ anti-phospho-STAT3 (Tyr-705) (D3A7) (#9145, from Cell Signaling Technology), anti-NFKBIA (L35A5, from Cell Signaling Technology), anti-NFKBIZ (TA502768S, from OriGene Technologies, Rockville, MD, USA), anti-α-tubulin (sc-8035, from Santa Cruz Biotechnology, Dallas, TX, USA), anti-β-actin (#4967, from Cell Signaling Technology) and anti-histone-H3 (Abcam, #ab1791). After blotting with the HRP-conjugated secondary antibody, the membrane was developed using a chemiluminescence HRP substrate kit (Thermo Scientific). Specificities of the anti-STAT3α and anti-STAT3β antibodies for the respective ΔS variants were demonstrated by western blotting of mini-proteins expressed for each of the four variants and comprising the SH2 module through the C-terminal of the variant.

### Quantitative RT–PCR

Total RNA was extracted using the RNeasy Plus Mini Kit (Qiagen, Valencia, CA, USA) according to the manufacturer's protocol, and quantified with a Nanodrop lite spectrophotometer (Thermo Scientific). For each sample, 2 μg RNA was reverse transcribed with a first-strand cDNA synthesis kit (Invitrogen, Carlsbad, CA, USA). All quantification was performed using an ABI Stepone Plus Real-Time PCR System (Foster City, CA, USA) in 96-well optical reaction plates. Primers were designed for absolute splice variant quantification, with reference to constructs of known concentration, as described previously.^21^ In addition, a primer pair recognizing a region present in all STAT3 splice variants (spanning exons 19 and 20) was used for relative qPCR, with GAPDH as a reference gene.^39^ By combining absolute and relative qPCR quantification data, mRNA levels were calculated: mRNA level=pan-STAT3 mRNA level (relative qPCR quantification) × STAT3 isoform percentage (absolute qPCR quantification).

### Statistical analysis

In single and combination STAT3 experiment, the rescue efficiencies of different STAT3 variant stable cell lines on day 12 were calculated: Rescue efficiency=(survival rate of STAT3 variant stable cell line−survival rate of EV)/(survival rate of STAT3Sα-C−survival rate of EV). Two-tailed Student's *t*-test and two-way ANOVA were performed to analyze statistical significance. *P*<0.05 and *P*<0.01 were used to show statistical significance throughout the article. Spearman's rank correlation coefficient was used to assess the correlation between rescue efficiency and expression of NFKBIA or NFKBIZ.
